# Hexaaqua­magnesium(II) bis­{5-[3-(1*H*-tetra­zol-5-yl)phen­yl]tetra­zolide} tetra­hydrate

**DOI:** 10.1107/S1600536812001250

**Published:** 2012-01-18

**Authors:** Cheng-Fang Qiao, Chun-Sheng Zhou

**Affiliations:** a Department of Chemistry and Chemical Engineering, Shaanxi Key Laboratory of Comprehensive Utilization of Tailing Resources, Shangluo University, Shangluo 726000, Shaanxi, People’s Republic of China

## Abstract

The asymmetric unit of the title compound, [Mg(H_2_O)_6_](C_8_H_5_N_8_)_2_·4H_2_O, contains one half of the centrosymmetric dication, one anion and two water mol­ecules. The Mg^II^ ion is coordinated by six water mol­ecules in a slightly distorted octa­hedral geometry. In the anion, the two five-membered heterocycles are twisted from the central benzene ring by 4.34 (11) and 3.20 (10)°. In the crystal, O—H⋯N, O—H⋯O and N—H⋯O hydrogen bonds generate a three-dimensional network.

## Related literature

For background to tetra­zole-containing compounds, see: Zhao *et al.* (2008 ▶). For related structures, see: Lü (2008 ▶); Kostakis *et al.* (2009*a* ▶,*b* ▶).
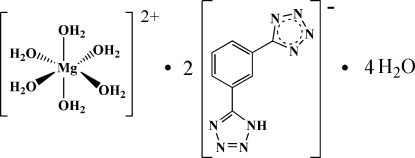


## Experimental

### 

#### Crystal data

[Mg(H_2_O)_6_](C_8_H_5_N_8_)_2_·4H_2_O
*M* *_r_* = 630.87
Monoclinic, 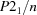

*a* = 7.3776 (12) Å
*b* = 16.038 (3) Å
*c* = 12.1787 (19) Å
β = 102.199 (2)°
*V* = 1408.5 (4) Å^3^
*Z* = 2
Mo *K*α radiation
μ = 0.14 mm^−1^
*T* = 296 K
0.31 × 0.24 × 0.09 mm

#### Data collection

Bruker APEXII CCD diffractometer
Absorption correction: multi-scan (*SADABS*; Bruker, 2008 ▶) *T*_min_ = 0.960, *T*_max_ = 0.987
6895 measured reflections
2496 independent reflections
2033 reflections with *I* > 2σ(*I*)
*R*_int_ = 0.023

#### Refinement

*R*[*F* ^2^ > 2σ(*F* ^2^)] = 0.043
*wR*(*F* ^2^) = 0.126
*S* = 1.06
2496 reflections
196 parameters
H-atom parameters constrained
Δρ_max_ = 0.36 e Å^−3^
Δρ_min_ = −0.46 e Å^−3^

### 

Data collection: *APEX2* (Bruker, 2008 ▶); cell refinement: *SAINT* (Bruker, 2008 ▶); data reduction: *SAINT*; program(s) used to solve structure: *SHELXS97* (Sheldrick, 2008 ▶); program(s) used to refine structure: *SHELXL97* (Sheldrick, 2008 ▶); molecular graphics: *ORTEP-3* (Farrugia, 1997 ▶); software used to prepare material for publication: *SHELXL97*.

## Supplementary Material

Crystal structure: contains datablock(s) I, global. DOI: 10.1107/S1600536812001250/cv5229sup1.cif

Structure factors: contains datablock(s) I. DOI: 10.1107/S1600536812001250/cv5229Isup2.hkl

Additional supplementary materials:  crystallographic information; 3D view; checkCIF report

## Figures and Tables

**Table 1:** Hydrogen-bond geometry (Å, °)

*D*—H⋯*A*	*D*—H	H⋯*A*	*D*⋯*A*	*D*—H⋯*A*
O5—H5*B*⋯N5^i^	0.86	2.02	2.876 (2)	172
O5—H5*A*⋯N7^ii^	0.84	2.11	2.921 (2)	164
O4—H4*B*⋯N6^i^	0.87	2.32	3.067 (2)	144
O4—H4*A*⋯N3^iii^	0.86	1.89	2.739 (2)	174
O3—H3*A*⋯N1^iv^	0.85	2.16	2.786 (2)	130
O2—H2*B*⋯O5^v^	0.87	1.93	2.787 (2)	172
O2—H2*A*⋯O5^iii^	0.85	1.90	2.737 (2)	173
O1—H1*B*⋯N2^iv^	0.85	1.99	2.821 (2)	164
O1—H1*A*⋯N4^iii^	0.85	2.09	2.880 (2)	156
N8—H8⋯O4^ii^	0.86	1.92	2.743 (2)	159

Symmetry codes: (i) 
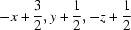
; (ii) 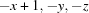; (iii) 
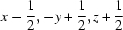
; (iv) 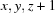; (v) 
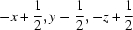
.

**Fig. 1.**
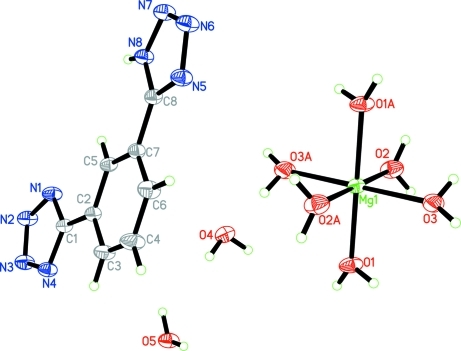
The content of asymmetric part of (I) showing the atomic numbering and 30% probabilty displacement ellipsoids [symmetry code: (A) -x + 1, -y, -z + 1].

## supplementary crystallographic information

### Comment

Tetrazole compounds have attracted a great deal of attention in the recent years because of their potential as functional materials (Zhao *et al.*, 2008; Kostakis *et al.*, 2009*a*,*b*). For an important tetrazole derivative, 5,5'-(1,3-phenylene)bis(1*H*-tetrazole), some interesting and multifunctional transition metal and rare earths metal complexes with it were reported (Lü, 2008; Kostakis *et al.*, 2009*a*,*b*). However, reports on alkaline earth metal compounds with it are very scarce. We report here the synthesis and crystal structure of the title compound (I).

The asymmetric unit of (I) consists of one half of an [Mg(H_2_O)_6_]^2+^ cation, one mono-deprotonated 5,5'-(1,3-phenylene)bis(1*H*-tetrazole) anion and two solvent water molecules (Fig.1). The Mg—O bond lengths range from 2.0425 (15) to 2.0793 (15) Å [mean value = 2.0654 (15) Å]. In the crystal structure, intermolecular O—H···N, O—H···O and N—H···O hydrogen bonds (Table 1) link cations, anions and water molecules into a three-dimensinal network. The crystal packing exhibits also intermolecular π-π stacking interactions between five-membered rings with centroid-centroid distances 3.6877 (6) and 3.7959 (6) Å, respectively.

### Experimental

A mixture of MgCl_2_.6H_2_O (0.0812 g, 0.4 mmol), NaOH (0.0160 g, 0.4 mmol), 5,5'-(1,3-phenylene)bis(1*H*-tetrazole) (0.0860 g, 0.4 mmol) and distilled H_2_O (8 ml) was sealed in a 15 ml Teflon-lined stainless steel vessel, which was heated at 413 K for 3 days. After the sample was cooled to room temperature at a rate of 5 *^o^*C/h, the colourless block crystals of (I) suitable for X-ray analysis were obtained.

### Refinement

All the H atoms attached to C and N atoms were placed geometrically (C—H = 0.93 and N—H = 0.86 Å) and refined as riding with *U*_iso_(H) = 1.2*U*_eq_(C,*N*). The water H atoms were located in difference Fourier maps and refined initially with restraints O—H = 0.85 (2) Å, however, in the last cycles of refinement, there were refined as riding, with *U*_iso_(H) = 1.5*U*_eq_(O).

### Crystal data

**Table:**

[Mg(H_2_O)_6_](C_8_H_5_N_8_)_2_·4H_2_O	*F*(000) = 660
*M**_r_* = 630.87	*D*_x_ = 1.488 Mg m^−^^3^*D*_m_ = 1.488 Mg m^−^^3^*D*_m_ measured by not measured
Monoclinic, *P*2_1_/*n*	Mo *K*α radiation, λ = 0.71073 Å
Hall symbol: -P 2yn	Cell parameters from 2462 reflections
*a* = 7.3776 (12) Å	θ = 3.0–26.7°
*b* = 16.038 (3) Å	µ = 0.14 mm^−^^1^
*c* = 12.1787 (19) Å	*T* = 296 K
β = 102.199 (2)°	Block, colourless
*V* = 1408.5 (4) Å^3^	0.31 × 0.24 × 0.09 mm
*Z* = 2	

### Data collection

**Table:**

Bruker APEXII CCD diffractometer	2496 independent reflections
Radiation source: fine-focus sealed tube	2033 reflections with *I* > 2σ(*I*)
graphite	*R*_int_ = 0.023
φ and ω scans	θ_max_ = 25.1°, θ_min_ = 2.1°
Absorption correction: multi-scan (*SADABS*; Bruker, 2008)	*h* = −6→8
*T*_min_ = 0.960, *T*_max_ = 0.987	*k* = −19→17
6895 measured reflections	*l* = −14→14

### Refinement

**Table:**

Refinement on *F*^2^	Primary atom site location: structure-invariant direct methods
Least-squares matrix: full	Secondary atom site location: difference Fourier map
*R*[*F*^2^ > 2σ(*F*^2^)] = 0.043	Hydrogen site location: inferred from neighbouring sites
*wR*(*F*^2^) = 0.126	H-atom parameters constrained
*S* = 1.06	*w* = 1/[σ^2^(*F*_o_^2^) + (0.0669*P*)^2^ + 0.5848*P*] where *P* = (*F*_o_^2^ + 2*F*_c_^2^)/3
2496 reflections	(Δ/σ)_max_ < 0.001
196 parameters	Δρ_max_ = 0.36 e Å^−^^3^
0 restraints	Δρ_min_ = −0.46 e Å^−^^3^

### Special details

**Table:**

Geometry. All e.s.d.'s (except the e.s.d. in the dihedral angle between two l.s. planes) are estimated using the full covariance matrix. The cell e.s.d.'s are taken into account individually in the estimation of e.s.d.'s in distances, angles and torsion angles; correlations between e.s.d.'s in cell parameters are only used when they are defined by crystal symmetry. An approximate (isotropic) treatment of cell e.s.d.'s is used for estimating e.s.d.'s involving l.s. planes.
Refinement. Refinement of *F*^2^ against ALL reflections. The weighted *R*-factor *wR* and goodness of fit *S* are based on *F*^2^, conventional *R*-factors *R* are based on *F*, with *F* set to zero for negative *F*^2^. The threshold expression of *F*^2^ > σ(*F*^2^) is used only for calculating *R*-factors(gt) *etc*. and is not relevant to the choice of reflections for refinement. *R*-factors based on *F*^2^ are statistically about twice as large as those based on *F*, and *R*- factors based on ALL data will be even larger.

### Fractional atomic coordinates and isotropic or equivalent isotropic displacement parameters (Å^2^)

**Table:**

	*x*	*y*	*z*	*U*_iso_*/*U*_eq_	
Mg1	0.5000	0.0000	0.5000	0.0304 (3)	
C1	0.7904 (3)	0.15136 (12)	−0.09794 (16)	0.0298 (4)	
C2	0.8281 (3)	0.10454 (12)	0.00838 (16)	0.0297 (4)	
C3	0.9335 (3)	0.14064 (13)	0.10478 (18)	0.0403 (5)	
H3	0.9753	0.1952	0.1023	0.048*	
C4	0.9768 (4)	0.09637 (14)	0.20399 (19)	0.0481 (6)	
H4	1.0476	0.1211	0.2679	0.058*	
C5	0.7649 (3)	0.02305 (12)	0.01326 (16)	0.0307 (4)	
H5	0.6933	−0.0016	−0.0505	0.037*	
C6	0.9152 (3)	0.01543 (14)	0.20876 (18)	0.0429 (5)	
H6	0.9451	−0.0143	0.2757	0.051*	
C7	0.8087 (3)	−0.02159 (12)	0.11354 (16)	0.0306 (4)	
C8	0.7505 (3)	−0.10822 (12)	0.12182 (16)	0.0316 (4)	
N1	0.6957 (3)	0.12118 (10)	−0.19549 (14)	0.0382 (4)	
N2	0.7003 (3)	0.18189 (11)	−0.27055 (14)	0.0413 (5)	
N3	0.7922 (3)	0.24562 (10)	−0.21978 (14)	0.0390 (4)	
N4	0.8508 (2)	0.22850 (10)	−0.11037 (14)	0.0366 (4)	
N5	0.7909 (3)	−0.15548 (10)	0.21375 (15)	0.0386 (4)	
N6	0.7168 (3)	−0.23122 (11)	0.18397 (16)	0.0433 (5)	
N7	0.6331 (3)	−0.23186 (11)	0.07931 (16)	0.0426 (5)	
N8	0.6528 (2)	−0.15522 (10)	0.03918 (14)	0.0350 (4)	
H8	0.6097	−0.1389	−0.0286	0.042*	
O1	0.5231 (2)	0.12872 (9)	0.51251 (12)	0.0429 (4)	
H1A	0.5065	0.1746	0.4769	0.064*	
H1B	0.5592	0.1400	0.5819	0.064*	
O2	0.2173 (2)	0.00747 (9)	0.46229 (14)	0.0488 (4)	
H2A	0.1668	0.0466	0.4914	0.073*	
H2B	0.1493	−0.0367	0.4605	0.073*	
O3	0.5006 (3)	−0.00392 (9)	0.67073 (12)	0.0502 (5)	
H3A	0.5486	0.0092	0.7382	0.075*	
H3B	0.4035	−0.0323	0.6713	0.075*	
O4	0.5005 (2)	0.14487 (9)	0.18614 (12)	0.0431 (4)	
H4A	0.4286	0.1770	0.2133	0.065*	
H4B	0.6097	0.1676	0.2042	0.065*	
O5	0.5296 (2)	0.37416 (9)	0.05607 (13)	0.0458 (4)	
H5A	0.5036	0.3291	0.0216	0.069*	
H5B	0.5769	0.3613	0.1249	0.069*	

### Atomic displacement parameters (Å^2^)

**Table:**

	*U*^11^	*U*^22^	*U*^33^	*U*^12^	*U*^13^	*U*^23^
Mg1	0.0341 (5)	0.0253 (5)	0.0291 (5)	−0.0006 (4)	0.0003 (4)	−0.0021 (4)
C1	0.0325 (10)	0.0248 (10)	0.0301 (10)	−0.0002 (8)	0.0018 (8)	−0.0012 (8)
C2	0.0327 (10)	0.0260 (10)	0.0294 (10)	0.0000 (8)	0.0039 (8)	−0.0004 (8)
C3	0.0515 (13)	0.0301 (11)	0.0355 (11)	−0.0112 (9)	0.0009 (10)	−0.0005 (9)
C4	0.0660 (15)	0.0393 (13)	0.0313 (11)	−0.0155 (11)	−0.0075 (11)	−0.0019 (10)
C5	0.0333 (10)	0.0275 (10)	0.0288 (10)	−0.0016 (8)	0.0011 (8)	−0.0033 (8)
C6	0.0571 (14)	0.0362 (12)	0.0303 (11)	−0.0057 (10)	−0.0023 (10)	0.0068 (9)
C7	0.0337 (10)	0.0251 (10)	0.0324 (10)	0.0011 (8)	0.0057 (8)	0.0011 (8)
C8	0.0355 (10)	0.0265 (10)	0.0317 (10)	0.0010 (8)	0.0047 (8)	0.0002 (8)
N1	0.0507 (11)	0.0283 (9)	0.0306 (9)	−0.0049 (8)	−0.0027 (8)	0.0021 (7)
N2	0.0546 (12)	0.0317 (10)	0.0320 (9)	−0.0031 (8)	−0.0036 (8)	0.0018 (8)
N3	0.0504 (11)	0.0303 (9)	0.0318 (9)	−0.0041 (8)	−0.0016 (8)	0.0032 (7)
N4	0.0479 (10)	0.0279 (9)	0.0305 (9)	−0.0054 (8)	0.0001 (8)	0.0024 (7)
N5	0.0485 (11)	0.0286 (9)	0.0355 (9)	−0.0012 (8)	0.0018 (8)	0.0040 (7)
N6	0.0545 (11)	0.0287 (10)	0.0432 (11)	−0.0044 (8)	0.0023 (9)	0.0043 (8)
N7	0.0517 (11)	0.0288 (10)	0.0445 (11)	−0.0041 (8)	0.0039 (9)	0.0003 (8)
N8	0.0444 (10)	0.0267 (9)	0.0317 (9)	−0.0027 (7)	0.0027 (8)	0.0012 (7)
O1	0.0627 (10)	0.0246 (8)	0.0331 (8)	0.0012 (7)	−0.0083 (7)	0.0011 (6)
O2	0.0354 (8)	0.0437 (10)	0.0649 (11)	−0.0002 (7)	0.0050 (8)	−0.0130 (8)
O3	0.0754 (12)	0.0427 (9)	0.0300 (8)	−0.0199 (8)	0.0052 (8)	−0.0035 (7)
O4	0.0500 (9)	0.0369 (9)	0.0406 (8)	0.0009 (7)	0.0055 (7)	−0.0070 (7)
O5	0.0583 (10)	0.0314 (8)	0.0428 (9)	−0.0052 (7)	0.0000 (8)	0.0009 (7)

### Geometric parameters (Å, °)

**Table:**

Mg1—O2	2.0425 (15)	C7—C8	1.464 (3)
Mg1—O2^i^	2.0425 (15)	C8—N5	1.332 (3)
Mg1—O1	2.0744 (14)	C8—N8	1.340 (3)
Mg1—O1^i^	2.0744 (14)	N1—N2	1.341 (2)
Mg1—O3	2.0793 (15)	N2—N3	1.308 (2)
Mg1—O3^i^	2.0793 (15)	N3—N4	1.340 (2)
Mg1—H3B	2.3995	N5—N6	1.350 (2)
C1—N4	1.334 (2)	N6—N7	1.294 (3)
C1—N1	1.335 (3)	N7—N8	1.342 (2)
C1—C2	1.472 (3)	N8—H8	0.8600
C2—C3	1.390 (3)	O1—H1A	0.8500
C2—C5	1.393 (3)	O1—H1B	0.8501
C3—C4	1.379 (3)	O2—H2A	0.8454
C3—H3	0.9300	O2—H2B	0.8653
C4—C6	1.381 (3)	O3—H3A	0.8500
C4—H4	0.9300	O3—H3B	0.8500
C5—C7	1.393 (3)	O4—H4A	0.8555
C5—H5	0.9300	O4—H4B	0.8695
C6—C7	1.389 (3)	O5—H5A	0.8375
C6—H6	0.9300	O5—H5B	0.8620
			
O2—Mg1—O2^i^	180.0	C2—C5—C7	120.11 (18)
O2—Mg1—O1	91.28 (6)	C2—C5—H5	119.9
O2^i^—Mg1—O1	88.72 (6)	C7—C5—H5	119.9
O2—Mg1—O1^i^	88.72 (6)	C4—C6—C7	119.99 (19)
O2^i^—Mg1—O1^i^	91.28 (6)	C4—C6—H6	120.0
O1—Mg1—O1^i^	180.0	C7—C6—H6	120.0
O2—Mg1—O3	90.74 (7)	C6—C7—C5	119.84 (19)
O2^i^—Mg1—O3	89.26 (7)	C6—C7—C8	118.06 (18)
O1—Mg1—O3	88.51 (6)	C5—C7—C8	122.08 (18)
O1^i^—Mg1—O3	91.49 (6)	N5—C8—N8	107.48 (17)
O2—Mg1—O3^i^	89.26 (7)	N5—C8—C7	125.53 (18)
O2^i^—Mg1—O3^i^	90.74 (7)	N8—C8—C7	126.96 (18)
O1—Mg1—O3^i^	91.49 (6)	C1—N1—N2	105.03 (16)
O1^i^—Mg1—O3^i^	88.51 (6)	N3—N2—N1	109.31 (16)
O3—Mg1—O3^i^	180.0	N2—N3—N4	109.65 (16)
O2—Mg1—H3B	74.4	C1—N4—N3	104.93 (16)
O2^i^—Mg1—H3B	105.6	C8—N5—N6	106.28 (17)
O1—Mg1—H3B	100.7	N7—N6—N5	110.72 (16)
O1^i^—Mg1—H3B	79.3	N6—N7—N8	106.59 (16)
O3—Mg1—H3B	20.3	C8—N8—N7	108.93 (16)
O3^i^—Mg1—H3B	159.7	C8—N8—H8	125.5
N4—C1—N1	111.08 (17)	N7—N8—H8	125.5
N4—C1—C2	124.57 (17)	Mg1—O1—H1A	145.5
N1—C1—C2	124.32 (18)	Mg1—O1—H1B	106.7
C3—C2—C5	119.16 (18)	H1A—O1—H1B	107.7
C3—C2—C1	119.90 (18)	Mg1—O2—H2A	117.9
C5—C2—C1	120.90 (17)	Mg1—O2—H2B	121.1
C4—C3—C2	120.65 (19)	H2A—O2—H2B	108.4
C4—C3—H3	119.7	Mg1—O3—H3A	150.7
C2—C3—H3	119.7	Mg1—O3—H3B	101.6
C3—C4—C6	120.2 (2)	H3A—O3—H3B	107.7
C3—C4—H4	119.9	H4A—O4—H4B	105.4
C6—C4—H4	119.9	H5A—O5—H5B	106.4

Symmetry codes: (i) −*x*+1, −*y*, −*z*+1.

### Hydrogen-bond geometry (Å, °)

**Table:**

*D*—H···*A*	*D*—H	H···*A*	*D*···*A*	*D*—H···*A*
O5—H5B···N5^ii^	0.86	2.02	2.876 (2)	172
O5—H5A···N7^iii^	0.84	2.11	2.921 (2)	164
O4—H4B···N6^ii^	0.87	2.32	3.067 (2)	144
O4—H4A···N3^iv^	0.86	1.89	2.739 (2)	174
O3—H3A···N1^v^	0.85	2.16	2.786 (2)	130
O2—H2B···O5^vi^	0.87	1.93	2.787 (2)	172
O2—H2A···O5^iv^	0.85	1.90	2.737 (2)	173
O1—H1B···N2^v^	0.85	1.99	2.821 (2)	164
O1—H1A···N4^iv^	0.85	2.09	2.880 (2)	156
N8—H8···O4^iii^	0.86	1.92	2.743 (2)	159

Symmetry codes: (ii) −*x*+3/2, *y*+1/2, −*z*+1/2; (iii) −*x*+1, −*y*, −*z*; (iv) *x*−1/2, −*y*+1/2, *z*+1/2; (v) *x*, *y*, *z*+1; (vi) −*x*+1/2, *y*−1/2, −*z*+1/2.
